# Primary Ovarian Angiosarcoma: A Case Report and Review of the Literature

**DOI:** 10.7759/cureus.110845

**Published:** 2026-06-14

**Authors:** Rajni Parmar, Vijay Yadav, Ankur Agarwal

**Affiliations:** 1 Histopathology, Oncquest Laboratories Ltd, Delhi, IND; 2 Surgical Oncology, Manipal Hospital, Jaipur, IND; 3 Laboratory Medicine, Manipal Hospital, Jaipur, IND

**Keywords:** angiosarcoma, clinical trials, immunohistochemistry, ovary, poor prognosis, rare site

## Abstract

Angiosarcomas are rare sarcomas with a poor prognosis, and treatment modalities are still being established. Cutaneous and soft tissue angiosarcomas are frequently reported; however, those occurring at rare sites have fewer published case reports and series. An unusual case was encountered at our institute: a 25-year-old woman with an ovarian tumor in which angiosarcoma was diagnosed in a background of teratoma. The details of surgery, gross appearance, histopathological findings, and immunohistochemistry (IHC) workup leading to the diagnosis are presented, along with a review of the current literature. The ease of reaching the histological diagnosis of angiosarcoma depends on the site of occurrence and degree of differentiation. Recent advances in therapeutics, along with emerging evidence from clinical trials, have demonstrated promising outcomes in the treatment of angiosarcoma. It is possible to reach an accurate diagnosis by keeping in mind its histological variability and key histologic features and by performing an appropriate IHC workup. Improving treatment results for patients with angiosarcoma involves a shift toward multimodal therapy and precision medicine, and recent breakthroughs offer hope for better outcomes.

## Introduction

Angiosarcomas represent only about 1.6%-2% of all soft-tissue sarcomas [1,2]. Angiosarcoma is a rare, highly aggressive form of cancer that originates in the endothelium of blood or lymphatic vessels. As blood vessels are present throughout the body, it can develop anywhere. While the exact cause is often unknown, several established risk factors and types exist. Primary cutaneous angiosarcoma most commonly appears on the scalp and face, typically affecting older adults. Radiation-induced angiosarcoma develops in areas of the body that have received previous radiation therapy, such as the chest wall after breast cancer radiation. Lymphedema-associated angiosarcoma develops in areas with chronic, long-term lymphatic swelling, often following mastectomy or lymph node removal. There are increasing worldwide publications of case reports of angiosarcoma occurring at other rare sites. One of these rare sites is the ovary. Angiosarcoma of primary ovarian origin was first described in 1931, and only a few case reports and small series have been published in the English-language literature. The development of a somatic malignancy in the form of a sarcoma is a well-known phenomenon in germ cell tumors. Malignant transformation of certain mesenchymal elements within teratomas and origin from primitive germ cells are some of the proposed theories that have been put forward in the literature [3-9]. It is important to keep in mind the possibility of angiosarcomas at rare sites, their histological variability, and key histologic features, and to perform an appropriate immunohistochemistry study to reach an accurate diagnosis. Angiosarcomas are aggressive neoplasms with a poor prognosis. Recently, a large cancer database analysis study identified survival and prognostic factors among patients with angiosarcoma [10]. Recent advances in therapeutics, along with emerging evidence from clinical trials, have demonstrated promising outcomes in the treatment of angiosarcoma [11]. We present a case of ovarian angiosarcoma that presented at our center.

## Case presentation

**Figure 1:**
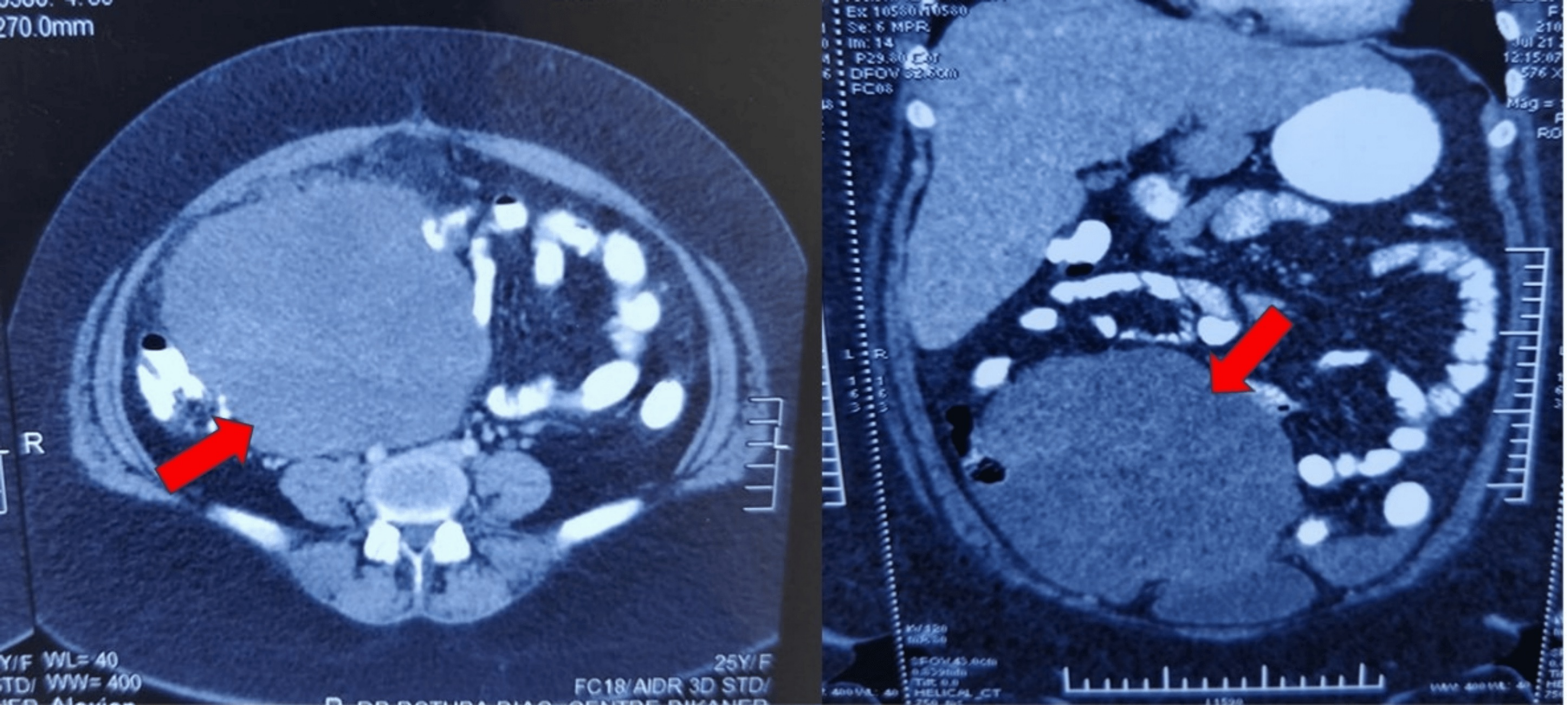
Contrast-enhanced computed tomography (CECT) of the abdomen. CECT of the abdomen showed a large, lobulated, relatively well-circumscribed cystic mass measuring approximately 128 × 150 × 178 mm on the right side of the abdomen and pelvis, extending inferiorly into the right adnexal region (red arrow).

A 25-year-old woman presented to the gastroenterology clinic with a two- to three-month history of abdominal pain, nausea, bloating, and intermittent constipation. Physical examination revealed a palpable lower abdominal mass. Subsequent ultrasound evaluation suggested the presence of an ovarian mass. She was then referred to the regional hospital, where further management was performed. Non-contrast computed tomography (NCCT), followed by contrast-enhanced computed tomography (CECT) of the abdomen performed on July 21, showed a large, lobulated, relatively well-circumscribed cystic mass measuring approximately 128 × 150 × 178 mm on the right side of the abdomen and pelvis, extending inferiorly into the right adnexal region. On post-contrast images, mild enhancement was seen with internal septations. No sizable solid component, calcific foci, or fat was noted. The right ovary was not separately visualized. The lesion was compressing and displacing adjacent structures, abutting the bowel loops, uterus, and urinary bladder. The adjacent omentum appeared mildly thickened (Figure 1).

After the radiological findings, her serum tumor marker levels were tested on July 27. Laboratory results showed that CA-125 was elevated at 164.1 U/mL. Serum urea, creatinine, serum beta-human chorionic gonadotropin (beta-hCG), and alpha-fetoprotein (AFP) were within normal limits (Table 1).

**Table 1:** Laboratory test results. CA-125: Cancer antigen 125; hCG: Human chorionic gonadotropin; U/mL: Units per milliliter; IU/mL: International units per milliliter.

Laboratory parameter	Result value	Reference range
CA-125	164.1 U/mL	0-35 U/mL
Serum beta-hCG	0.199 mIU/mL	<5 mIU/mL
Alpha-fetoprotein (AFP)	2.88 IU/mL	0-40 IU/mL

**Figure 2:**
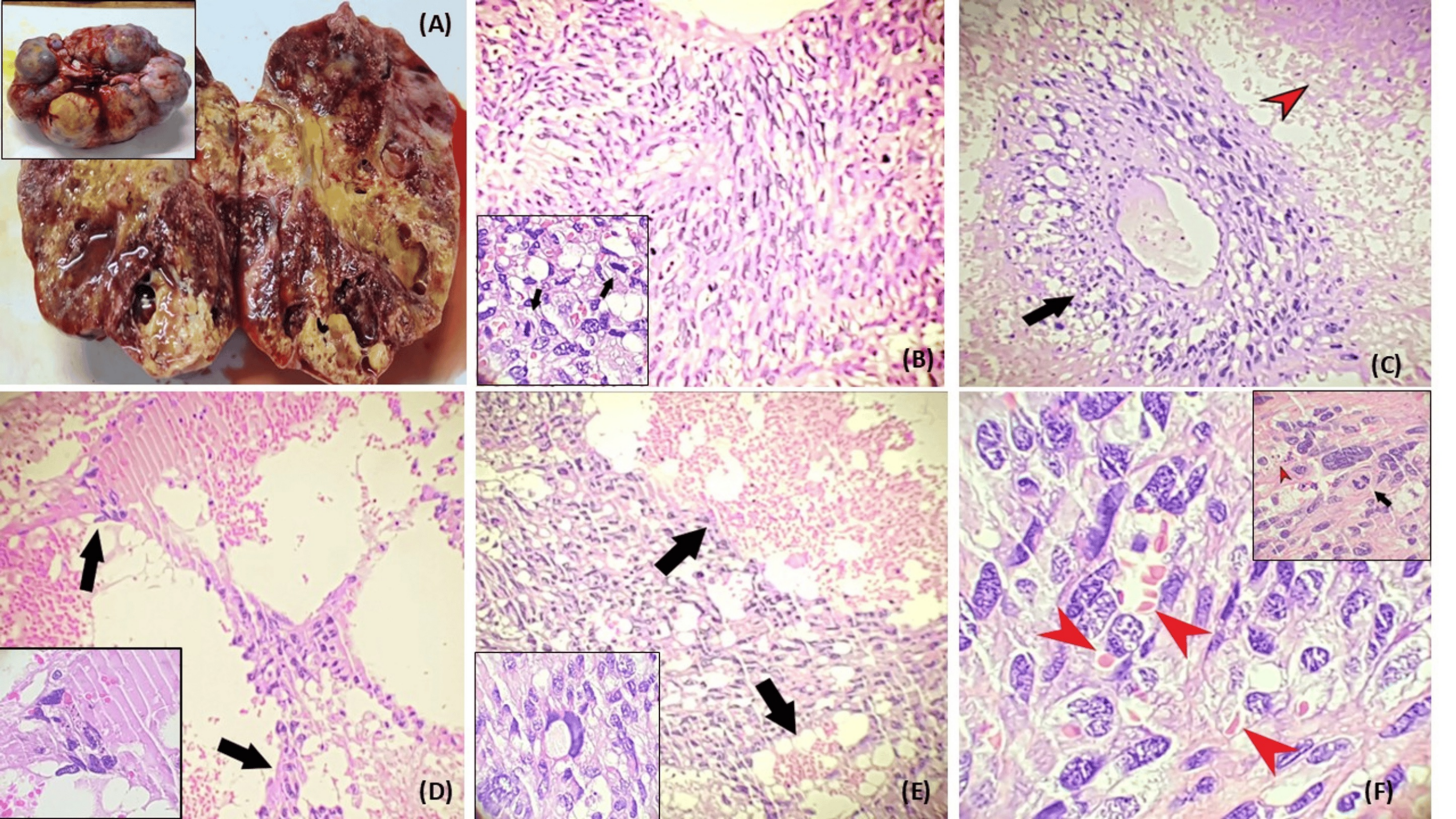
Ovarian tumor histopathology. (A) Grossly, the ovarian tumor had a fleshy brown cut surface with areas of hemorrhage; the inset shows the bosselated external surface. (B) Atypical spindle cells arranged in a fascicular pattern (H&E, ×100), with the inset showing frequent mitoses (black arrows) (H&E, ×400). (C) Large areas of necrosis (red arrowhead), with preserved tumor cells in areas surrounding blood vessels (black arrow) (H&E, ×200). (D, E) Wide vascular channels lined by atypical tumor cells (black arrow) (H&E, ×200), with the inset showing these atypical cells at higher magnification (H&E, ×400). (F) Tumor cells showing intracytoplasmic lumina containing RBCs (red arrowheads), with the inset showing pleomorphic tumor cells displaying mitoses (black arrow) and apoptosis (red arrowhead) (H&E, ×400).

Exploratory laparotomy was performed the next day, July 28, through a midline incision, and a large mass involving the right ovary with ascites was found. The frozen section was reported as suggestive of a germ cell tumor. The omentum was found to be adherent to the tumor. The left ovary was normal appearing. Right salpingo-oophorectomy and omentectomy were performed. Ascitic fluid was sent for examination and was reported to be negative for malignant cells. Gross examination of the right ovarian specimen showed a bulky, large encapsulated mass measuring 21 × 18 × 12 cm. The capsule was intact. The outer surface was bosselated. The cut surface was fleshy, brown to pink, friable, and showed areas of hemorrhage and necrosis (Figure 2A). Histopathology was reported as immature teratoma of the ovary, grade 3. The patient improved symptomatically and was discharged. The paraffin blocks were then sent to a reference laboratory for a second opinion and immunohistochemistry workup. The initial H&E stain showed that the tumor had large areas of necrosis with preserved tumor cells in areas surrounding blood vessels. In the preserved areas, the tumor was composed of atypical spindle cells arranged in a predominantly fascicular pattern and, in many areas, showed a sponge-like appearance due to the formation of small, irregular vascular spaces containing red blood cells. Several wider vascular channels were also seen, lined by atypical endothelial cells. In more solid areas, the tumor cells displayed intracytoplasmic vascular lumina formation. Many of these lumina contained red blood cells. The lesional cells showed moderate to marked nuclear atypia with the presence of multinucleated tumor cells. The nuclei were spindle shaped, the chromatin was dark and clumped, and the nuclear margins were irregular. Nucleoli were variable. Many mitotic figures, including atypical ones, were seen (Figures 2B-2F).

**Figure 3:**
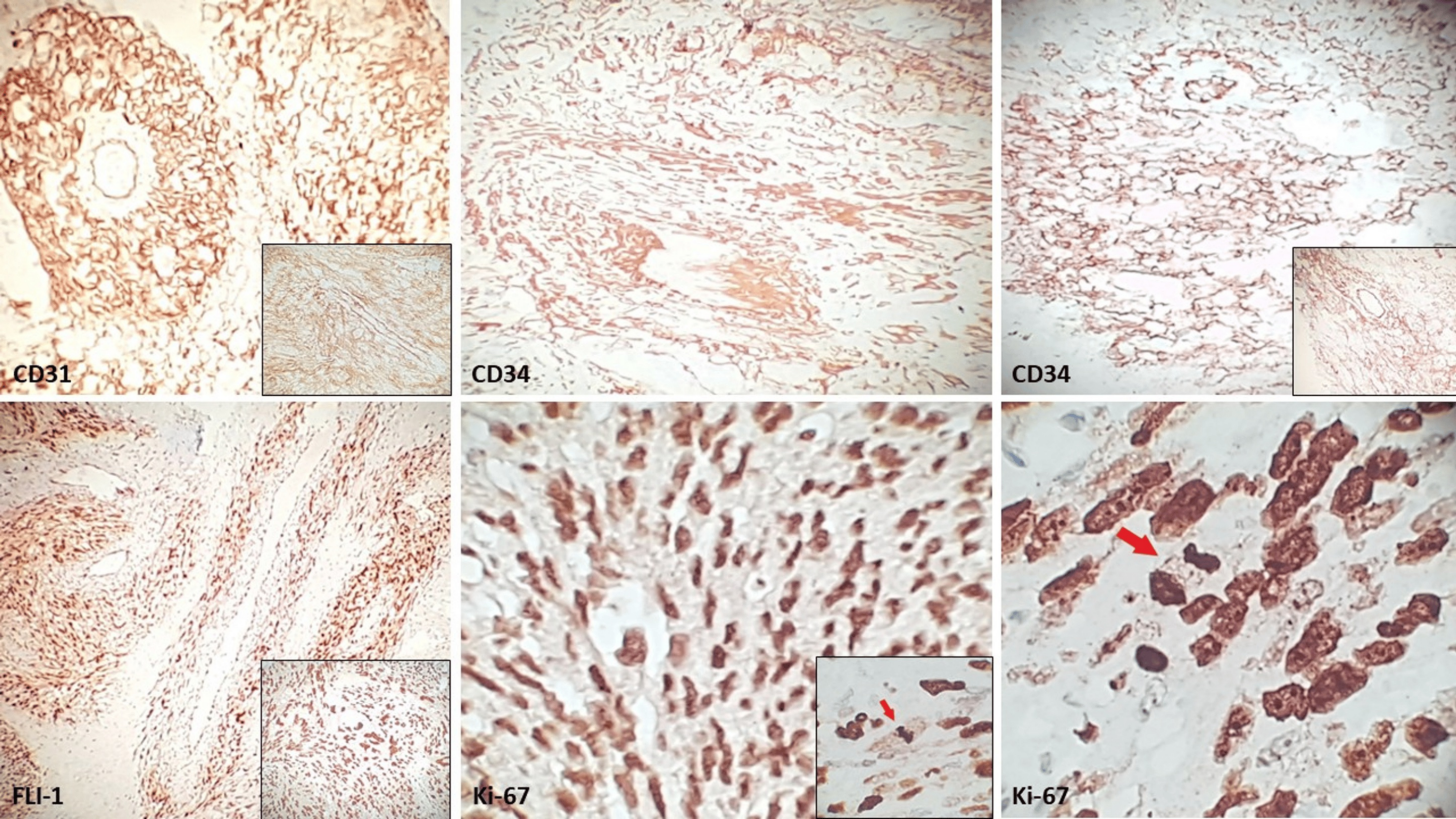
Ovarian tumor immunohistochemistry. CD31 (top left, with inset) shows strong and diffuse positivity in the tumor cells (IHC, ×200). CD34 (top middle and top right, with inset) shows positivity in many tumor cells (IHC, ×200). FLI-1 (bottom left, with inset) shows strong and diffuse positivity in the tumor cells (IHC, ×200). Ki-67 (bottom middle, with inset, and bottom right) shows strong positivity, with a proliferation index of 80% (IHC, ×400). Mitoses are highlighted by red arrows (IHC, ×400). CD31: Cluster of differentiation 31; CD34: Cluster of differentiation 34; FLI-1: Friend leukemia integration 1 transcription factor; IHC: Immunohistochemistry.

Immunohistochemical staining was strongly and diffusely positive for CD31 and FLI-1. Staining for CD34 and SMA was also positive in many tumor cells. Immunohistochemical staining for CK, EMA, desmin, and CK7 was negative. Ki-67 staining showed a proliferation index of 80% (Figure 3). The histopathological study confirmed the diagnosis of angiosarcoma, FNCLCC grade 3.

The patient recovered well in the postoperative period. After the immunohistochemistry reports and discussion by the tumor board, a whole-body PET scan was performed. The PET scan was suggestive of a few iliac lymph nodes, with no visceral or osseous metastasis. As per the tumor board discussion, adjuvant chemotherapy was planned in the form of ifosfamide and Adriamycin. She was treated with adjuvant chemotherapy with ifosfamide and Adriamycin and was alive two years later.

## Discussion

Angiosarcomas occur rarely and mainly in the skin and soft tissues, with the head and neck being the most common site. Systemic organ angiosarcomas are less common, and those of the ovary are very rare.

Clinical presentation and radiological features

Ovarian angiosarcomas present with abdominal pain, distension, and bowel movement changes [3]. The imaging features in the ovary have been described as a large solid or solid-cystic mass. In the ovary, angiosarcomas are commonly associated with other neoplasms, the most common being teratoma. Therefore, imaging in these cases may show a complex mass of mixed composition [5].

Histopathological diagnosis

The ease of reaching the histological diagnosis of angiosarcoma depends on the site of occurrence and differentiation. At clinically common sites and with vasoformative morphology, such as low-grade tumors, it may be easy to diagnose. However, at rare sites and/or with poor differentiation, diagnosis may be challenging. Cases may be missed altogether if not suspected.

Traditionally, angiosarcomas were graded as well differentiated, moderately differentiated, and poorly differentiated based on vasoformation, mitosis, necrosis, and nuclear atypia. A well-differentiated tumor can resemble a benign hemangioma, and poorly differentiated angiosarcoma can have a solid pattern that may be indistinguishable from a sarcoma. The epithelioid variant can resemble carcinoma or melanoma [12]. The differential diagnosis under consideration would also vary depending on the site at which it presents.

The ovarian angiosarcoma had an undifferentiated spindle cell morphology; hence, the differentials ranged from leiomyosarcoma, endometrial stromal sarcoma, solitary fibrous tumor, and metastatic gastrointestinal stromal tumor (GIST) to malignant melanoma. Immunohistochemistry was required to reach the diagnosis. Negative immunoreactivity for SMA, desmin, pan-CK, CD10, ER, STAT6, CD117, DOG1, S100, SOX-10, and HMB45 ruled out these differentials. Careful search identified a vasoformative pattern, and the tumor was immunopositive for CD31, CD34, and FLI-1, confirming the diagnosis.

A well-differentiated hemangioma-like pattern would need to be differentiated from benign hemangioma and vascular granulation tissue. Histological features to consider would include pleomorphism of endothelial cells, hyperchromatic nuclei, irregular nuclear outlines, hobnail protrusion of endothelial cells into vascular lumina, and atypical mitotic figures. Endothelial immunohistochemical markers, including CD31, CD34, and FLI-1, would be positive in the tumor cells. Of note, immunohistochemical markers of endothelial origin, such as CD31, ERG, and FLI-1, which are used to diagnose a malignant vascular neoplasm, are also positive in benign lesions and normal vasculature and hence cannot be used to differentiate between them [12]. The histological features, in combination with IHC markers, would help confirm the diagnosis.

It has recently been described that MYC immunohistochemical expression and amplification detected by FISH can differentiate secondary angiosarcomas from atypical vascular lesions but not from primary angiosarcomas [12].

Angiosarcomas are not likely to be the first differential consideration in the ovary; however, with increasing knowledge of their occurrence, histopathologic features, and immunohistochemical expression, they can be diagnosed accurately at these rare sites.

Notably, with increasing experience and the development of new markers, the variability of endothelial marker expression has now come to light. Immunohistochemistry with endothelial markers shows weak and focal expression in poorly differentiated areas of angiosarcomas, whereas it shows stronger and more diffuse expression in vasoformative areas [13]. There can also be immunopositivity for epithelial differentiation markers, specifically in epithelioid variants [14]. Therefore, a panel of markers should be applied to accommodate this overlap. The histological features should be closely assessed, and a combination of these diagnostic tools will help to arrive at the correct diagnosis.

Prognosis

The survival of patients with angiosarcoma is poor. The age spectrum of patients with ovarian angiosarcoma has a wide range, including children and young adults. In a series of seven patients with an age range of 20-32 years, two patients with stage I disease survived 5.5 and 9 years, respectively (Nielsen GP et al.) [6]. Younger patients similar to ours have also been reported; for example, a 19-year-old patient survived for one year (Davidson B et al.) [8]. In a study of angiosarcomas at multiple sites, including 4,537 cases extracted from the Surveillance, Epidemiology, and End Results (SEER) database, Zhang C et al. found that the overall 1-, 2-, and 5-year survival rates were 55.2%, 41.0%, and 26.3%, respectively. Compared with angiosarcoma at other sites, patients with angiosarcoma in rare organs had the worst survival. They also found that tumor size ≥5 cm, no surgery, regional and distant tumors, high grade, and age older than 69 years were negatively associated with survival [10]. Fayette J et al., in their retrospective analysis of 161 angiosarcomas, reported that angiosarcomas are a heterogeneous group of tumors with distinct clinical behavior, with a strong impact of disease site on outcome. In their retrospective study of breast, skin, and soft tissue angiosarcomas, the 5-year overall survival rate was reported as 43% (95% CI, 33%-53%) [15]. Merfeld E et al., in their study of prognostic features of angiosarcoma in 65 cases, found that angiosarcomas that arose as a result of previous radiation therapy had worse outcomes compared with primary angiosarcomas [16]. More recently, the 5-year overall survival for gynecologic angiosarcomas was reported as 27% (SE, 8%) [17]. Ren S et al., in their study of survival predictors of metastatic angiosarcoma, reported that chemotherapy, radiotherapy, and tumor size were independent predictors of overall survival [18]. In a later study, Weidema ME et al. reported remarkably poorer overall survival for primary angiosarcoma compared with secondary angiosarcoma among 479 confirmed angiosarcoma patients [19].

Treatment

The choice of treatment for angiosarcoma has limited consensus. Surgical resection with adjuvant radiotherapy remains the cornerstone of treatment for patients with localized angiosarcomas. A multimodal approach is preferred for advanced-stage disease. Fang C et al., in their retrospective study of 128 angiosarcoma cases, showed that primary surgery improved survival in localized disease, with adjuvant radiotherapy enhancing local control but not overall survival. Their study underscored the heterogeneity of angiosarcoma across different sites, emphasizing the role of surgery for localized angiosarcoma, with chemotherapy being the mainstay for advanced cases [20]. There are promising trials with anti-angiogenic agents. Advances in molecular characterization may lead to better therapeutic targets. Clinical trials for angiosarcoma focus on immunotherapy, targeted therapies, and novel combinations for advanced/metastatic disease. Key studies involve checkpoint inhibitors, such as nivolumab, ipilimumab, and avelumab, and anti-angiogenic agents, such as pazopanib and regorafenib, to improve outcomes. Recent research highlights combined approaches for better efficacy in this rare, aggressive tumor.

## Conclusions

Angiosarcomas are rare, aggressive tumors with a historically poor prognosis. Although therapeutic modalities remain in flux, an expanding body of recent literature highlights ongoing efforts to refine management protocols and improve survival rates. The possibility of this diagnosis should be kept in mind when evaluating poorly differentiated spindle cell/epithelioid cell neoplasms at visceral and other rare sites, and a wide panel of immunohistochemical markers should be performed to cover possible differential diagnoses. It is possible to reach an accurate diagnosis by keeping in mind their histological variability and key histologic features and by performing an appropriate immunohistochemistry workup. Improving treatment results for patients with angiosarcoma involves a shift toward multimodal therapy and precision medicine, and recent breakthroughs offer hope for better outcomes.
